# Exploring practices of Dermatologists in Ethical Dilemmas in Pakistan: A narrative analysis

**DOI:** 10.12669/pjms.342.14328

**Published:** 2018

**Authors:** Moizza Tahir, Rahila Yasmeen, Rehan Ahmed Khan

**Affiliations:** 1 Dr. Moizza Tahir, MCPS(Med), FCPS(Derm), MHPE. Associate Professor, Department of Dermatology, Combined Military Hospital/Quetta Institute of Medical Sciences Quetta, Pakistan; 2 Prof. Dr. Rahila Yasmeen, BDS, DCPS-HPE. HOD of Medical Education, Director MHPE Program, Associate Director (RARE/ORIC), Faculty of Health &Medical Sciences, Riphah International University, Islamabad, Pakistan; 3 Prof. Dr. Rehan Ahmed Khan, MBBS(Pak), FCPS (Pak), FRCS (Ire), M-HPE, M.Sc.HPE, PhD(Scholar) Assistant Dean of Medical Education, Professor of Surgery, Islamic International Medical College.

**Keywords:** Dermatoethics, Dermatology and ethical dilemmas, Cosmetology and ethical dilemmas

## Abstract

**Background and Objective:**

Specialists in dermatology come across ethical issues in their practice. The topic is subjective so dialogue and reflection is required. The main objective of this study was to explore how dermatologists deal with ethical dilemmas in their clinical practice.

**Methods:**

This was a qualitative narrative analysis. It was conducted by involving dermatologists working in tertiary care hospitals across Pakistan from January to June 2017. Open ended semi structured in-depth interviews of twelve dermatologists were recorded and transcribed manually through transcribers verbatim. Thematic interactional analysis was done by NVivo 11.

**Results:**

Ethical dilemmas were narrated. Thematic analysis showed that compromises in standard medical and ethical practices were made on academics and training. Ethics were left to individual choice. Consultation of patients suffered due to quality of patient physician relationships and breeched patient’s confidentiality. In cosmetic dermatology unrestrained role of media, injudicious procedures and improper counseling created difficulties. Pharmaceuticals relation revolved around conflict of interest. In sexually transmitted diseases disclosure were difficult due to sociocultural limitations. In teledermatology practices patient’s confidentiality and consent were compromised while consultation remained challenging for dermatologists being visual only.

**Conclusion:**

Dilemmas appearing in everyday life needs peer discussion, reflections and protocols that should be role modeled.

## INTRODUCTION

‘As physicians, most of us consider ourselves as ethical in everything we do. Until I looked deeper into medical ethics, I would have said that I was ethical. As it turns out, I am not as ethical as I thought I was’ (Karen Scully ).^1^

Experience, knowledge, and being a good person do not promise that physicians can recognize or resolve ethical dilemmas.^2^ Lasers, biological therapies, surgical interventions, phototherapies and aesthetic practices have inherent ethical issues. Training in ethics and professionalism is the formal requirement of accreditation bodies of American and Canadian residency programmes. Content on ethics however is not prescribed, dialogue and reflection on ethical controversies are encouraged.^3^ Literature search shows dearth of dermatology oriented articles on ethics.^4^

Ethical principles are needed to clarify rights of patient.^5^ Pakistan Medical and Dental Council, the regulatory body of all medical practioners has documented a common code of ethics for undergraduate curriculum.^6^ HCEC has formulated guidelines to assist faculty in introducing ethics to undergraduate students.^7^ Its application needs assurance.^8^ The topic is subjective so it is important to have dialogue and reflection on ethical concerns in dermatology.^9^ Developments in medical ethics require development at state level.^10^ Thus, there is a need to conduct a qualitative study to explore how physicians respond when faced with ethical dilemmas.^11^

There is dearth of published literature on ethical dilemmas in dermatology, although similar studies exist in other fields of medicine in literature.^12^,^13^ The present study explores dermatologists dealing with ethical dilemmas.

## METHODS

Theoretical and conceptual frame work of study was based on principles of medical ethics; autonomy, justice, beneficence and non-maleficence.^5^ It was a qualitative narrative analysis best suited for bioethics.^14^,^15^ The study was approved by ethical review committee.

Study was conducted from January to June 2017. Study population included twelve (12) dermatologists across teaching hospitals of Pakistan. This sample size was enough to saturate data.^16^ Purposive convenience sampling technique was employed as key informant dermatologists in teaching and enough clinical experience were selected.^17^ Inclusion criteria included dermatologist with PMDC recognized qualifications. Dermatologists with qualification in medical education were preferred. Dermatologists with non-clinical qualifications were excluded. Data was collected by semi structured open ended audio recorded interviews once, on pre scheduled appointment after informed consent. Interview questions were developed after literature review.

Questions were validated by two subject experts and three medical educationists. Interview format and sample dilemma were e-mailed to participants after informed consent.

After pilot interview prompts were added. The first question ‘Do you face any ethical problems in the practice of dermatology?’ was to build rapport and open discussion. This was followed by question ‘What are your ethical concerns in the practice of dermatology?’ Probe for narration of experience or relevant story was given. Interview question were deciphered into themes, as interviews continued new themes emerged sideways (Table-I). Data was analyzed by thematic interactional analysis in NVivo 11. It was transcribed through transcribers verbatim in English. Codes were merged into six major themes. Identification of narrative to characterize key aspect as a whole or partial was done. Narratives were analyzed at “manifest level”.^18^

**Table I:** Developments of themes.

**Themes acknowledged from literature review**
Revolutionary therapies, Dermato-cosmetology, Patient physician relationship,
Teledermatology, Clinical photography, Relation with Pharmaceutical
**Questions about themes**
What are your ethical concerns in dermatology?
What are your ethical concerns in cosmetology?
**Deciphering of questions into themes**
Compromises in standard medical and ethical practices, Cosmetology related dilemmas
**Novel themes appeared sideways with old themes**
Ethical Dilemmas of Consultation, STD, Teledermatology, Pharmaceuticals

Each interview had duration for an hour. Reflexivity was adopted by mutual collaboration and minimal interruption. Interview transcripts were shared with participants for feedback and for credibility. Environmental triangulation was done by recruiting participants from four provinces of Pakistan.^19^ Theory triangulation was achieved by sharing transcripts for interpretation with another FCPS qualified dermatologist working as registrar in government setup.^19^ Peer debriefing was done by an evaluator, bachelors in civil engineering for feedback on interpretations. There was consensus on coding, themes and subthemes. Transferability is allowed by details of context in methodology for readers.^20^

## RESULTS

Of twelve dermatologists, 7(58.3%) were male and 5(41.6%) were female, aged between 37 and 58 years (mean 48.25 years). All of them were practicing in tertiary care hospitals and were involved in teaching of under and post graduate students. Three (25%) of them were from Karachi, 4(33.3%) from Rawalpindi, 2(16.6%) from Quetta, 1(8.3%) from Lahore and 2 (16.6%) were from Peshawar. All participants were FCPS in dermatology. Additionally 2(16.6%) of them were qualified in MHPE; 9(75%) had certification course in Medical education. Two of them were Programme directors, four were head of departments, three were associate professors, other was assistant professor and one was registrar dermatology.

### Abbreviation used

**FCPS:** Fellow of College of Physicians and Surgeons Pakistan

**MCPS:** Member of College of Physicians and surgeons of Pakistan

**MHPE:** Masters in health Profession Education

**PMDC:** Pakistan Medical and Dental council

**CPSP:** College of Physicians and surgeons Pakistan

**HEC:** Higher Education Commission

**HCEC:** Health Care Ethics Committee

**NBC:** National Bioethics Committee

**STD:** Sexually transmitted disease

**FDA:** Food and Drug Administration

**NVivo:** Qualitative data analysis computer software package

Thematic interactional analysis lead to six themes representing major ideas, supported by sub themes representing allied perspectives.

## DISCUSSION

Participant dermatologists in our study shared difficult situations. Most of them had faced some ethically difficult situation in their clinical practice. Nine out of 12 dermatologists mentioned compromises in standard medical ethical practices. This emerged as new theme, dermatology curriculum doesn’t have structured component of ethics as laid down by other curriculum.^21^ Peer group discussion, opinions of people outside medicine and contact to skilled mentors can be used for ethics education through structured training programmes.^22^ HCEC ethical guidelines can be used for structured post graduate residency programmes in dermatology.^7^

Dilemmas related to ethics of consultation were due to prescriptions and counseling. Polypharmacy in prescription is associated with a higher likelihood of inappropriate medications, as consistent with other studies.^23^ Counseling about drugs is encouraged by pharmacist internationally.^24^

Ten respondents mentioned dilemmas in cosmetology practices. Individuals are exposed to images of “ideal” bodies portrayed by media.^25^ This is reflected in the choice and demands for treatment.^26^ Bioethics education produces substantial variations in thinking and actions.^27^ Patient participation in making a choice and skill of qualified dermatologists would be the best course of action.

Nine dermatologists related ethical dilemmas in relations to pharmaceuticals. Accepting free drug samples was considered appropriate but even these samples were found to be influential on prescriptions.^28^ Central collections of free drug samples should be encouraged for distribution to deserving patients. To avoid any conflict of interest, healthcare professionals for the larger interest of the profession should stay cautious.^29^

Six participants found difficulty in disclosure of STDs and considered culture as limitation. Counseling remained challenging.^29^,^30^ Compromises in confidentiality is a source of distress. Cultural differences should be considered for disclosures and to improve treatment compliance.^31^

**Table-II:** Codes for sub theme of Injudicious Procedure in theme Cosmetology related dilemmas.

Injudicious Procedures	Harm to patient
	Free experimentation
	Injudicious cosmetic procedures
	Conflict of interest
	Autonomy on cosmetic concerns
	Non FDA approved drugs
	Heightened expectations
	Undue demand of Inj Glutathione

**Table-III:** Theme with verbatim.

**1. Compromises in standard medical and ethical practices**	
Sub-theme	Verbatim
Ethics as individualized practice	Unfortunately ethics is personal.
Compromised teaching	Most of practicing dermatologists are not interested in teaching undergraduates and postgraduates. Most of the time…ah… focus is on service demands and not on academics.
	There should be separate time allocation for academics for clinical teachers.
**2. Ethical dilemmas of cosmetology**	
Unrestrained role of media	I think practicing dermatology has become difficult with time and it’s mainly the role of media.
	We guide patients about their demands.
Injudicious procedures	Small blemishes are given undue importance for treatment. Patients are reassured wrongly about procedures and time, while blemishes prevail.
	Just counseling is required.
Counseling	Most of the ladies ask for injection Glutathione to get their skin shade better but I tell them that it may not be a right choice.
	Can you narrate any example?
	Yeah….there was a relative of my colleague and was lactating mother, I told her that glutathione would harm her baby; she is an educated lady and just changing the complexion, would not improve the quality of life.
	I think that doctor’s counseling does help.
**3. Ethical dilemmas related to pharmaceuticals**	
Conflict of interest	Why do the doctors advise patients to get medicine from a peculiar pharmacy? Write medicines that suit to the disease, but the idea is to increase sale of the pharmaceutical companies
	I don’t recommend any specific pharmacy.
**4. Ethical dilemmas in consultation**	
Polypharmacy	Patients are with loads of medication for single problem.
	A patient with fungal infection presents with list of 10 drugs you name and find it there.
Patients’ obliviousness to their prescriptions	Most of the doctors do not inform patient about prescribed medicines. He has no clue of what has been given.
	I really spend more than 10 to 15 minutes on a single patient. Most of the time, I tell them it is their rights to know about medicine, diagnosis and any other thing.
Patients confidentiality disregarded Doctor patient relation need crystallization	A young girl of Turner syndrome was seen by me. I asked for karyotyping. Report was brought by grandfather. I told him about diagnosis of Turner syndrome.
	Few days later father visited with same reports I said, “I have already told grandfather about karyotype report.”
	He said now it would be a problem for us.
	I realized then that we should talk to the parents and no one else about such diagnosis.
	I must tell my patient that this is my time cost this much profit I am going to take for this medicine procurement. It must not be hidden in between patient and physician. The most important thing is again trust, patient trusts the doctor and …Doctor must ….maintain that trust.
**5. Disclosures in sexually transmitted disease remained challenging (STD)**	
A patient came to me with primary syphilis. I catered him and told him that his wife needs treatment. I convinced her that she should get the injection although I tried not to reveal. The natural question of his wife was how did my husband acquire this? Although there was no answer but to satisfy her I told her that there were so many reasons in which a person can get this disease, however, once the disease is there, likely it may spread.	
P: Why not truth?	
That’s a dilemma I cannot tell the truth even now.	
**6. Teledermatology practices patients consent is marginalized**	
1.Patient discussing problem with doctor is not fine as one is not sure from picture.	
I tell them to meet because whatever consultation you give, you become responsible for it. If it goes wrong patient’s stance “you told to do it so we did it”.	
So I do not believe in consultation like that.	
2. Main sore point in teledermatology is patient’s secrecy and privacy. We are not …ah…respecting patient’s privacy. We take snapshots of patients without informing them and that we are using them in open forums …	
I think… informed consent must be taken from every patient for any aspect of medicine, discussion, research or record, or to see improvement in patient condition.	

Dermatology is well suited to telemedicine for discussion with colleague. Consent for images must be taken. Clarification should be made about the use of an image. Appropriate safety measures are required in all digital communications. Images must be removed after securing them to patient health records. Breaching image privacy is an emerging medico-legal risk.^32^ Government pilot projects of teledermatology would be useful in meeting health care needs for remote areas of Pakistan.

### Limitations

Paralinguistic and field notes were not recorded as interviews were audio recorded. Data triangulation was a limitation due to researcher location.

## CONCLUSION

Ethical dilemmas in dermatology are encountered in clinical practice. Peer discussion, reflections and protocols should be role modeled. Ethics education should be implemented through structured programmes as per guidelines of NBC.^7^

## RECOMMENDATION

In the light of contemporary trends, there is need to include short courses of ethics in post graduate residency programmes of dermatology.
